# High-resolution 7T fMRI reveals the visual zone of the human claustrum

**DOI:** 10.1162/imag_a_00327

**Published:** 2024-10-24

**Authors:** Adam Coates, David Linhardt, Christian Windischberger, Anja Ischebeck, Natalia Zaretskaya

**Affiliations:** Institute of Psychology, University of Graz, Graz, Austria; BioTechMed, Graz, Austria; High-Field MR Center, Center for Medical Physics and Biomedical Engineering, Medical University of Vienna, Vienna, Austria

**Keywords:** claustrum, 7 tesla, ultra-high field, fMRI, visual, auditory

## Abstract

The claustrum is a thin grey matter structure located between the insular cortexand the putamen. The function of the claustrum is largely unknown with diversehypotheses ranging from multisensory integration and consciousness to attentionand cognitive control. Much research on the function of the claustrum relies oninvasive techniques in animal models, as the claustrum’s uniquely thinshape makes it difficult to image non-invasively in human subjects. In thecurrent proof-of-concept study, we used high-resolution ultra-high field (7Tesla) functional magnetic resonance imaging (fMRI) to measure activity in thehuman claustrum during the processing of naturalistic stimuli. We presentedshort video clips as visual only, auditory only, or audiovisual conditions whileparticipants performed a central fixation task. We found distinct visualresponses in both the left and the right claustrum at a consistent spatiallocation across participants, hemispheres, and sessions. We also founddeactivations in response to auditory stimulation. These deactivations wereconfined to the right claustrum and did not overlap with visual activity. Thedeactivation in response to auditory stimulation demonstrates the complexity ofthe claustrum’s functional organization and suggests functionaldifferentiation within the claustrum. This is the first study to demonstratesensory-specific effects within the human claustrum. It opens the possibilityfor studying the claustrum’s role in higher-level aspects of sensoryprocessing in humans.

## 1 Introduction

The claustrum is a thin bilateral subcortical structure that is situated between theputamen and the insula. It has reciprocal connections with most cortical andsubcortical brain areas, which suggest its important role in higher-level processing(Edelstein & Denaro, 2004;Jackson et al., 2020;J. B. Smith et al., 2020;Torgerson et al., 2015;Torgerson& Van Horn, 2014). The claustrum has also been shown to have ahigh density of serotonin 2a receptors (5-HT_2A_), the primary targets ofpsychedelic drugs (McKenna & Saavedra,1987;Nichols, 2016). Pathology ofthe claustrum has been linked to several neuropsychiatric disorders such asschizophrenia (Cascella et al., 2011), autism(Wegiel et al., 2014), andattention-deficit hyperactivity disorder (ADHD) (Dickstein et al., 2006). Despite these unique and intriguing propertiesand a clear clinical relevance, the function of the claustrum remains a rarelyinvestigated topic.

Several hypotheses have been proposed about the claustrum’s role in cognitionand brain functioning. Although the original suggestions of it being the site ofconsciousness (Crick & Koch, 2005) didnot find much empirical support (Bickel &Parvizi, 2019;Chau et al., 2015;Duffau et al., 2007), recent research inrodents clearly points to the claustrum’s role in attention and cognitivecontrol (Goll et al., 2015;White et al., 2018). For example, the claustrum was shownto inhibit activity to irrelevant sensory stimuli, making the animal more resilientto distraction (Atlan et al., 2018).Additionally, connections from the anterior cingulate cortex (ACC) to the claustrumwere found to be critical when mice are engaged in a cognitively challenging task(White et al., 2018). Furthermore, theclaustrum is thought to be involved in coordinating slow-wave cortical activity(Narikiyo et al., 2020), and theclaustrum’s homolog in reptiles has recently been shown to play a role ingenerating slow-wave sleep (Norimoto et al.,2020). In sum, the range of possible claustrum functions is quitediverse, end the evidence supporting them relatively scattered. Furthermore, due tothe differences in overall brain anatomy and cognitive abilities across the animalkingdom (Baizer et al., 2014;Croxson et al., 2018), it remains unclear to which extentanimal findings are generalizable to humans. Human studies on the function of theclaustrum are critical for understanding its contribution to higher cognitivefunctions.

A well-documented aspect of the claustrum’s functional organization is theexistence of sensory zones (Olson & Graybiel,1980;Remedios et al., 2010;Torgerson & Van Horn, 2014) withcorresponding topographically organized claustrocortical connections (Fernández-Miranda et al., 2008;Pathak & Fernandez-Miranda, 2014a;Smythies et al., 2012). Specifically, auditory,visual, and somatosensory zones have been described in the claustrum, with neuronspreferring sensory input of the corresponding modality. In the macaque, distinctauditory and visual zones have been described, with the auditory zone located moredorsally and visual zone more ventrally (Gattass etal., 2014;Remedios et al., 2010).The claustrum’s thin shape and its location deep within the brain arechallenging for conventional neuroimaging in human participants and until now theexistence of the sensory zones of the human claustrum has not been tested.

In this proof-of-concept study, we utilized ultra-high field 7T fMRI to determinewhether it is possible to elicit measurable visual and auditory responses in thehuman claustrum. We presented naturalistic visual, auditory, and audiovisual stimulito participants in a 7T fMRI experiment. We followed a similar design toRemedios et al. (2010), who also presentednaturalistic videos and found modality-specific zones in the claustrum; we thereforeexpected to find these modality-specific zones within the human claustrum, in whichthe visual zone would be located more ventrally and the auditory zone moredorsally.

## 2 Materials and Methods

The study’s design and methods were preregistered prior to conducting theexperiment on AsPredicted.org (https://aspredicted.org/hj4sr.pdf). Code and data are available on theOpen Science Framework (https://osf.io/7ebm2/).

### 2.1 Participants

Sixteen healthy participants were recruited to take part in this study. The exactnumber of participants was determined during preregistration and was informed byprevious investigations utilizing 7 Tesla fMRI that focused on subcorticalstructures and visual perception with a sample size of 6–8 participants(Denison et al., 2014;Poltoratski et al., 2019). One participanthad to be excluded due to poor data quality in the functional scans. Therefore,we had a total sample size of 15 (mean age = 24.60 years, SD =3.33 years, 11 females and 4 males, 14 right-handed). Participants had normal orcorrected-to-normal visual acuity, had no history of neurological impairments,and were not taking any medication at the time of participation. Allparticipants gave written informed consent prior to participation. The study wasapproved by the ethics committee of the Medical University of Vienna and wasconducted in accordance with the Declaration of Helsinki. Participants receivedmonetary reimbursement for their participation.

### 2.2 Experimental design

Subjects viewed visual, auditory, and audiovisual naturalistic stimuli with anoverlaid central fixation point. The center fixation point changed color every500 ms between red and nine different shades of green in a pseudorandom order inwhich we removed consecutive duplicate colors. The participant’s task wasto fixate on the central fixation point and to respond with a button press whenthe fixation point changed to the color red. The center fixation task ensuredthat participants were paying attention to the stimuli throughout the experimentand fixated their gaze at the center of the screen. Stimuli were presented usingPsychoPy v2021.2.3 (Peirce et al., 2019)software on a MacBook pro 13” running macOS 12 Monterey. Visualstimulation was projected on an MRI-compatible rear projection screen using anXGA VPL F X 40 projector (Sony Group Corporation, Minato, Tokyo, Japan). Thedisplay was situated inside the scanner bore, which participants viewed througha mirror attached to the head coil at a 45° angle. The display-to-mirrordistance was about 148 cm. Video stimuli were displayed at the full size of theMRI-compatible display (46.5 × 37 cm) at a 16:9 aspect ratio andsubtended 17.5° × 14° visual angle with a fixation pointsituated in the center of the screen measuring 4 mm in diameter with a visualangle of 0.15°. Auditory stimulation was delivered using passivenoise-cancellation S15 MR compatible in-ear earphones (Sensimetrics Corporation,Woburn, MA, USA). Prior to the beginning of the first functional run, asoundcheck was carried out to ensure that a sufficient level of loudness wasachieved despite the ongoing scanner noise during image acquisition. Thisrequired fMRI dummy scanning while participants listened to some audio andprovided feedback to increase or decrease the volume accordingly.

Stimuli consisted of naturalistic video scenes with a superimposed centralfixation point. Naturalistic stimuli have been shown to elicit visual andauditory responses in the primate claustrum (Remedios et al., 2010). The videos were selected from the websitePexels (https://www.pexels.com/), a digital media sharing website. All stimulimaterials used in this experiment were published to Pexels under a free-to-useand free-to-modify license (CC0, creative commons zero license). We used 48video stimuli of different species of animals or natural scenery, and we ensuredthat the videos contained motion such that they would not be perceived as stillimages (for an example of the stimuli, seehttps://osf.io/7ebm2/,“Example Stimuli”). The original video clips were modified usingFFmpeg version 2022-07-18-git-cb22d5ea3c-full_build-www.gyan.dev (FFmpeg, 2022) as follows. Each of the 48selected videos were first cut down to a length of 15 seconds and then modifiedtwice to create a visual only and an auditory only version. For both theaudiovisual and auditory only conditions, the audio was normalized, such thatthe maximum sound volume for each clip did not exceed a max value of 0 dB. Thesemodifications yielded a total of 144 unique 15-second clips each belonging toone of the three stimulus types, corresponding to the three experimentalconditions: audiovisual (AV), visual only (V), and auditory only (A).

To ensure consistency of our findings within individuals, each subject took partin two scanning sessions taking place on 2 separate days, with a mean timebetween session 1 and session 2 of 10.67 days (SD = 6.39 days). Eachsession contained 6 runs, and in each run 24 trials with stimulation werepresented to participants. Each trial belonged to one of the three experimentalconditions, with the order of conditions pseudorandomized and counterbalancedusing a first-order counterbalanced condition sequence, to minimize trialhistory effects (Brooks, 2012). On every7th trial, a baseline (no stimulus) was presented, resulting in a total of 28trials per run (seeFig. 1a, for an exampleof the presentation order). Each trial (including baseline) lasted 15 seconds.Each functional run lasted 420 seconds and participants took part in sixfunctional runs per session for a total on-task scan time of 42 minutes. Two ofthe pilot sessions included in the analysis only involved a single session with4 runs, and 1 pilot participant carried out 3 sessions resulting in 11 runs. Thepilot sessions were not included in the session-to-session consistencyanalysis.

**Fig. 1.**
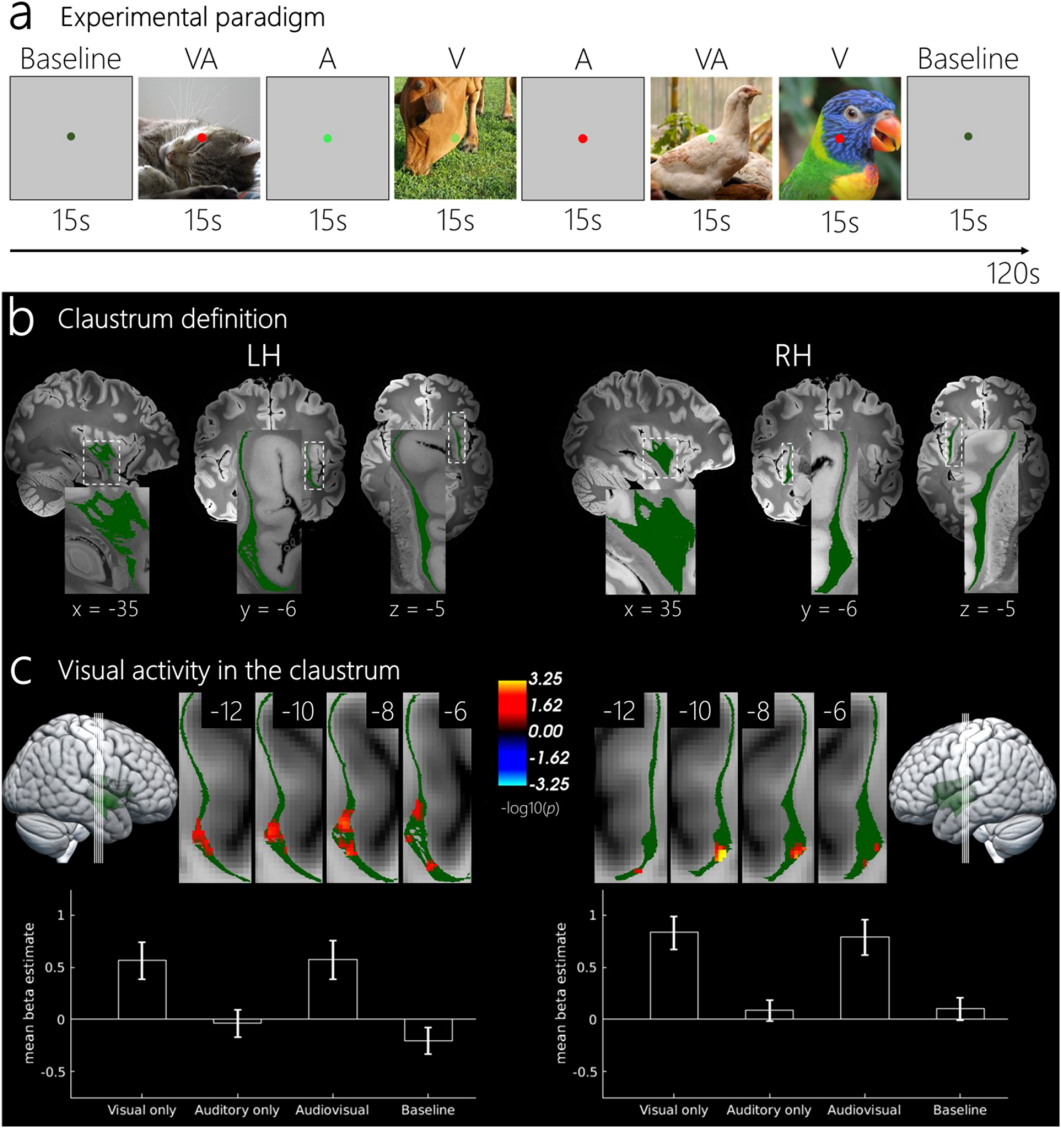
(a) An example of a typical condition sequence within a run. Eachcondition lasted for 15 seconds. The alternation of one baselinefollowed by six stimulus trials was repeated four times in each of thesix runs for each session. Participants were instructed to fixate on thecentral fixation point and to respond when the point changed to thecolor red. (b) Right and left claustrum label overlaid with theultrahigh-resolution MRI image (Edlow etal., 2019), which was used to identify the claustrum (Coates*&*Zaretskaya, 2024). Coordinates are shownin MNI space. (c) Visually evoked activity for the left and rightclaustrum. Four coronal slices represent the size and location of theclusters which are corrected for multiple comparisons at acluster-forming threshold of p < 0.01 and a cluster-wise p-valueof p < 0.05. Coordinates are shown in MNI space. Slices arezoomed-in on the claustrum ROI and do not represent the acquired fieldof view (seeSupplementary Fig. 1for the fields of view of everysubject). Bar plots represent the mean beta estimates for each conditionwithin the significant voxels. Error bars represent standard error ofthe mean (SEM).

### 2.3 MRI acquisition

#### 2.3.1 Functional MRI acquisition

MRI data were acquired using an ultra-high field 7 Tesla Siemens MAGNETOMscanner (Siemens Healthineers, Erlangen, Germany) using a 32-channel headcoil (Nova Medical, Wilmington, MA, USA). Blood oxygen level dependent(BOLD) contrast was obtained by using the gradient-recalled echo-planarimaging (GE-EPI) sequence. We acquired 37 sagittal slices at 1.34 mm× 1.34 mm resolution (slice thickness = 0.8 mm; TR =2000 ms; TE = 23 ms; FA = 62°, GRAPPA accelerationfactor = 2). The parameters deviated slightly in one of the sessionsof one pilot participant (TR = 2500 ms, 47 sagittal slices). Thisresulted in a partial brain coverage of either the left or right claustrum,depending on the participant (seeSupplementary Fig. 1for the acquired fields of viewof each subject). The EPI slice orientation and anisotropic voxel size werechosen to minimize blurring and maximize resolution along the left-rightdimension, where the claustrum is thinnest (Zaretskaya & Polimeni, 2016). The right claustrum wasscanned in six of the participants, and the left claustrum was scanned innine of the participants (including three pilot participants). Each run ofthe functional scan took 420 seconds to complete, yielding 220 volumes perrun. In addition to the main experimental runs, two additional volumes withidentical parameters and opposite phase-encoding directions were acquiredfor subsequent susceptibility distortion correction.

#### 2.3.2 Anatomical MRI acquisition

Anatomical images were acquired using a T1-weighted MP2RAGE sequence (Marques et al., 2010) with 0.75 mmisotropic voxel size (matrix size: 320 × 300 slices: TR = 4300ms; TE = 2.27 ms; FA = 4°, TI1 = 1000 ms, TI2= 3200 ms, GRAPPA R = 2). The total time taken to complete theMP2RAGE scan was 530 seconds. Anatomical scans were obtained during both thefirst and second scanning sessions except for pilots with one session andfor the pilot with three sessions (we used two of the anatomical scans forthe latter).

### 2.4 Statistical analysis

#### 2.4.1 MRI data preprocessing

The preprocessing of the anatomical scans consisted of the followingsteps.

Each of the two anatomical scans were first processed with the“presurfer” tool (https://github.com/srikash/presurfer) to remove extracerebralnoise from the MP2RAGE image by utilizing a bias-corrected second inversionof the MP2RAGE acquisition. After this, the two T1-weighted scans wereco-registered using the robust registration method (Reuter et al., 2010), which is part of theFreeSurfer package, and averaged to produce one final structural image persubject. This image was passed to the CAT 12.8.1 toolbox (Dahnke & Gaser, 2017) in SPM12 (7771, 13Jan., 2020) (http://www.fil.ion.ucl.ac.uk/spm/, 2011) (Penny et al., 2011) running on MATLAB R2019b(MathWorks, 2019). This was doneto create a high-quality brain mask by concatenating the white matter (WM)and grey matter (GM) segmentations. Each subject’s structural imagewas then used to perform cortical surface reconstruction usingFreeSurfer’s recon-all stream (Daleet al., 1999;Greve &Fischl, 2009) at native resolution (Zaretskaya et al., 2018), substitutingFreeSurfer’s auto-generated brain mask with the CAT12-derived brainmask.

Preprocessing of functional data involved motion correction, distortioncorrection, co-registration with the structural image, resampling to theanatomical image, and normalization to MNI space. The software used for eachof the functional preprocessing steps is listed inTable 1below.

**Table 1.** Steps involved in the preprocessing of functional data.

Step	Software	Command	Reference
Motion correction	AFNI (AFNI version 20.2.11) in FreeSurfer 7.1.0	mc-sess	Cox (1996) ( http://afni.nimh.nih.gov/afni/ )
Distortion correction	FSL 6.0.4	fslroifsl-mergetopupapplytopup	Andersson et al., (2003) ; Jenkinson et al., (2012) ( https://fsl.fmrib.ox.ac.uk/fsl/docs/#/diffusion/topup/users_guide/index?id=running-topup )
Cortical surface reconstruction	Freesurfer 7.1.0 recon-all	recon-all	Dale et al., (1999) ( https://surfer.nmr.mgh.harvard.edu/fswiki/recon-all )
Functional-structural coregistration	FreeSurfer 7.1.0 BBregister	mktemplate-sessbbregister	Greve and Fischl (2009) ; Fischl (2012) ( https://surfer.nmr.mgh.harvard.edu/fswiki/bbregister )
Upsampling to anatomical image	FreeSurfer 7.1.0	mri_vol2vol	Greve and Fischl (2009) ( https://surfer.nmr.mgh.harvard.edu/fswiki/mri_vol2vol )
Normalization to MNI space	(Presurfer, CAT12 (in SPM12)	matlabbatch.spatial.normalise.write	Dahnke and Gaser (2017) ( https://neuro-jena.github.io/cat/index.html )

First, the images were corrected for subject motion using the AFNI 3dvolregalgorithm that is implemented as part of FreeSurfer’s functionalanalysis stream (FSFAST). After this, the functional images were correctedfor susceptibility distortions due to magnetic field inhomogeneities usingFSL’s “*topup”*and“*applytopup”*(Andersson et al., 2003). Distortion-correctedfunctional images were co-registered to the averaged anatomical scan usingboundary-based registration with 6 degrees-of-freedom (Greve & Fischl, 2009). We then resampledthe functional data to the resolution of the structural scan (isotropicvoxel size 0.75 mm) using FreeSurfer’s“*mri_vol2vol”*command. This was done tobring the data from the two sessions into the same space in order to conductthe joint first-level analysis of both sessions. Normalization of thefunctional data to MNI space was performed by applying the nonlineartransformation derived from the CAT12 toolbox during anatomical processing.MNI-space data were additionally smoothed by convolving each volume with a3D Gaussian kernel with a full width at half maximum (FWHM) of 1.5 mm usingFreeSurfer’s “*mri_fwhm”*command.Smoothed data were used for the group voxel-wise GLM analysis. Unsmootheddata were used for individual subject analysis (reporting individual peakactivity of each subject and session-to-session consistency of voxelselectivity).

#### 2.4.2 Claustrum definition

To determine whether activations we observed in the functional experiment arelocated within the claustrum, it was necessary to accurately define the leftand the right claustrum in each individual. Standard atlases either do notcontain a claustrum label (Fischl et al.,2002,^2004^;Rolls et al., 2020) or label only thedorsal claustrum part (Ewert et al.,2018). Therefore, we manually labeled the left and rightclaustrum twice in an ultra-high resolution (0.1 mm isotropic) 7Tpost-mortem brain dataset, which is available in MNI space (Edlow et al., 2019). The union of the left andright claustrum label was then resampled to the anatomical space for use asa mask for statistical inference in group analysis and as masks for plottingthe individual subject data, as described in our previous study (Coates & Zaretskaya, 2024).

To ensure that the claustrum label derived from MNI space did not include anyof the neighboring structures in individual subject space (e.g., insula,putamen), we projected the MNI-space label into each subject’sindividual space by applying the inverse transformation generated by CAT12.FreeSurfer’s cortical-subcortical segmentation (aseg.mgz) was thenused to visually check whether any of the claustrum voxels overlap withstructures other than voxels labeled as white matter (because the claustrumlabel is not available in FreeSurfer 7.1.0). We found that often the putamenand insula labels overlapped with the claustrum label. However, a closervisual inspection of the correspondence between native T1-weighted imagesand generated labels showed that this was due to poor automatic segmentationof the insula and putamen rather than the inaccuracy of the claustrum label.Poor segmentation of the putamen and insula in FreeSurfer has been shownpreviously (Perlaki et al., 2017;Yaakub et al., 2020), and islikely to be caused by the partial volume effects between these structuresand the claustrum’s gray matter (Tianet al., 2020). As we did not find any overlap between theclaustrum label and the gray matter of the putamen or insula in T1-weightedimages, claustrum labels were unedited in all subjects.

#### 2.4.3 Individual-level analysis

Individual-level analysis was performed using FreeSurfer’s FSFASTusing a standard GLM approach with Visual (V), auditory (A), audiovisual(AV), and baseline conditions as regressors of interest, which wereconvolved with the canonical hemodynamic response function. In addition,run-specific offsets, scanner drifts (modeled with a quadratic polynomialterm), and the first 4 timepoints (to ensure that the scanner reachedmagnetic equilibrium) were modeled as nuisance regressors. To identifyvoxels within the claustrum that showed preference for either visual orauditory stimuli, we compared the beta estimates for the correspondingregressors, yielding the following contrasts: V − baseline and A− baseline. To identify voxels responding to both modalities, we alsocalculated a multisensory contrast. FollowingNoppeney (2012), we looked for a super-additiveeffect of the multisensory condition by calculating the contrast (AV+ baseline) − (A + V). A GLM fit and contrastcalculation was first performed individually for each session. The result ofeach session was then combined by carrying out a subject-level fixed-effectsGLM analysis for each contrast.

#### 2.4.4 Group analysis

For the second-level group analysis, we used the contrast estimates from theindividual-level GLMs to perform one-sample t-tests for nonzero effectsusing the random-effects GLM. Since the visual and auditory conditions areexpected to activate a small part of the whole claustrum, we corrected theresults for multiple comparisons within the claustrum label usingcluster-wise permutations (Greve &Fischl, 2018). We carried out 1000 permutations using acluster-forming threshold of p < 0.01 and a cluster significancelevel of p < 0.05 (Hagler et al.,2006) for each contrast. Since the left and the right claustrumdata cannot be combined in this approach, group analysis was performedtwice, once for subjects with the left claustrum scans (N = 9) andonce for subjects with the right claustrum scans (N = 6).

#### 2.4.5 Session-to-session consistency analysis

In order to determine whether the visual and auditory responses within theclaustrum of each individual appear consistently at the same location acrossboth session 1 and session 2, we performed an analysis in which we used datafrom either session to define a region of interest (ROI) and data from thecontrary session to measure the responses in that ROI. For example, tomeasure the visual response in session 1 we used session 2 to define thevisually responsive voxels (contrast “V − baseline”, p< 0.05 uncorrected) and then extracted the average contrast estimatesfrom these voxels in session 1. This procedure was repeated with session 2by defining visually responsive voxels using data from session 1. The sameanalysis was performed for auditory responses. The whole procedure yielded 4values for each of the 12 subjects: visual activations in session 1 and 2and auditory activations in session 1 and 2. Additionally, we calculatedsession-to-session consistency for auditory deactivations in session 1 and 2for the six subjects with only the right claustrum scanned. Subjects witheither their left or right claustrum scanned were pooled together.Session-to-session consistency was assessed by testing the ROI responsesagainst zero using a one-sample t-test.

#### 2.4.6 Control analysis of auditory cortex activity

Since we did not find auditory activations within the claustrum, we wanted toensure that the auditory stimuli used in the experiment were sufficient toactivate the auditory cortex. To achieve this, we defined voxelscorresponding to the transverse temporal gyrus (Heschl’s gyrus) inevery participant from the automatic segmentation generated by theFreeSurfer recon-all stream, for example, aparc+aseg.nii.gz (Dale et al., 1999;Fischl et al., 2002) using the“*mri_extractlabel”*command. Using theROI, we extracted average beta estimates for each condition and compared theauditory condition with baseline.

#### 2.4.7 Behavioral analysis

To ensure that the participants maintained stable gaze fixation throughoutthe experiment, we examined behavioral responses of each participant. To dothis, we examined a time period between 0 and 2000 ms after the onset ofeach red color to determine whether a response was given. A response made inthis time window was classified as a “hit.” We removedaccidental duplicate responses and kept only the first response participantsmade. We then calculated the percentage of hits$\left(\frac{numberofcorrectresponses}{numberoftotalredfixations}\times 100\right)$and mean reaction time for each participant. For two participants weencountered a technical issue with the button response box that failed torecord responses during two runs of the first session. Therefore, for thesetwo participants behavioral analysis was performed with the remining fourunaffected runs only. Moreover, analysis of behavioral responses for thefirst pilot subject was not possible due to a technical issue with fixationcolor changes not being logged by the script. Since we did record buttonpresses from this subject and the number of recorded presses was in therange of the remaining participants, it is unlikely that the participant didnot perform the task properly. We thus report behavioral performance for 14out of 15 subjects.

## 3 Results

Our aim in the current study was to determine whether high-resolution 7T fMRI wouldallow us to identify visual and auditory sensory zones of the human claustrum, andwhether these or any other regions of the claustrum show signs of audio-visualintegration. To test this, we presented participants with naturalistic video clipscontaining visual only, auditory only, or audiovisual information (seeFig. 1afor an example), while measuring fMRIactivity in either the left or the right claustrum. To unambiguously assignfunctional activations to the claustrum, we manually labeled the left and rightclaustrum in an ultrahigh-resolution post-mortem MRI image that was mapped to MNIspace (seeFig. 1b).

### 3.1 Visual responses within the human claustrum

**Fig. 2.**
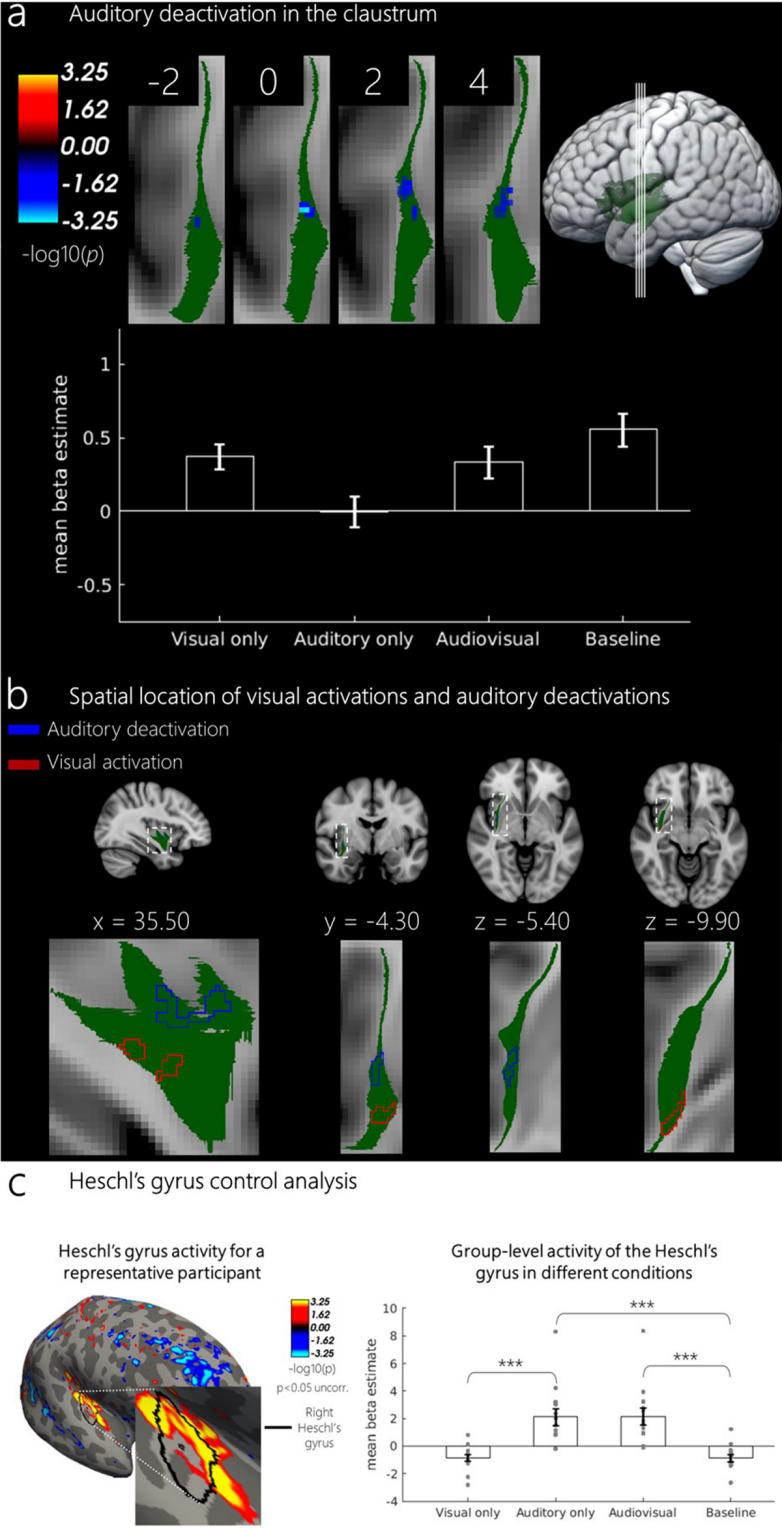
(a) Deactivations evoked by the auditory stimuli in the right claustrum.The reported cluster is corrected for multiple comparisons at acluster-forming threshold of p < 0.01 and a cluster-wise p-valueof p < 0.05. Coordinates represent the center of mass for thecluster. Bar plot represents the mean beta estimates for eachexperimental condition within the significant voxels. Error barsrepresent SEM. Slices are zoomed-in on the claustrum ROI and do notrepresent the acquired field of view (seeSupplementary Fig.1for the fields of view of every subject). (b) Righthemisphere with the clusters for the visual activations and auditorydeactivations shown as outlines to demonstrate that the clusters arespatially non-overlapping. (c) An example of a singleparticipant’s reconstructed surface (subject 2) showingsignificant auditory activation in the first session (p < 0.05,uncorrected) in the right Heschl’s gyrus. Bar plot represents thegroup-level mean beta estimates for the Heschl’s gyrus for eachexperimental condition (N = 15). Individual subject valuesrepresent an average activity of two sessions. Error bars represent SEM.***p < 0.001.

We performed a group analysis across all voxels within the claustrum for the leftand right claustrum separately, comparing responses of each unisensory conditionwith baseline. Our analysis revealed 2 significant clusters of voxels thatresponded stronger to visual stimulation compared to baseline in the leftclaustrum (most significant voxel in cluster 1; z-statistic = 3.3, p< 0.001, d = 1.7, MNI x= -32.2, y= -3.75, z=-12, cluster size = 132.9 mm^3^, most significant voxel, cluster2; z-statistic = 3.2, p < 0.01, d = 1.6, MNI x=-37.5, y= -1.5, z= -18.8, cluster size = 21.9mm^3^). We also found 1 significant cluster in the right claustrumthat responded stronger to visual stimulation compared to baseline (mostsignificant voxel in cluster; z-statistic = 3.9, p < 0.001, d= 4.4, MNI x= 32.2, y= -5.25, z= -11.2, cluster size= 52.3 mm^3^) (Fig. 1c). Todemonstrate that the group results originate from the claustrum and do notresult from the averaging of neighboring structures such as the putamen andinsula, we provide unmasked statistical maps of individual subjects overlaid ontheir individual anatomical images inSupplementary Figure 2.

We then ensured that significant visual activity observed in the claustrum at thegroup level is present in each individual subject at a similar spatial locationin unsmoothed data. To do this, we analyzed session 1 and session 2 of eachsubject together and then extracted MNI coordinates of the most significantvoxel for the visual versus baseline comparison. Peak coordinates of eachsubject as well as the mean and SD are shown inTable 2.Supplementary Figure 3shows the number of subjects with significantvisual claustrum activity for every voxel.

**Table 2.** Visual activations in individual subjects.

Subject	Hemisphere	-log10(p)	df	MNI coordinates		
				x	y	z
Pilot 1 ^a^	LH	4 ***	2113	-35.2	-4.5	-8.25
Pilot 2 ^b^	LH	2.9 **	849	-33.8	-4.5	-10.5
Pilot 3 ^b^	LH	3.4 ***	849	-30.8	-0.75	-12
Subject 1	RH	3.4 ***	2550	33.8	-9.75	-7.5
Subject 2	RH	3.8 ***	2550	33.8	-6.75	-9
Subject 3	RH	3.8 ***	2550	35.2	-10.5	-6
Subject 5	LH	8.6 ***	2550	-33.8	-9	-8.25
Subject 6	LH	5 ***	2550	-35.2	-4.5	-14.2
Subject 7	RH	3.75 ***	2550	36.8	2.25	-18.8
Subject 8	LH	14 ***	2550	-35.2	-10.5	-6.75
Subject 9	RH	12.9 ***	2550	34.5	-10.5	-6
Subject 10	RH	3.14 ***	2550	33.8	-6.75	-9
Subject 11	LH	3.83 ***	2550	-36	-15	-5.25
Subject 12	LH	5.68 ***	2550	-37.5	-16.5	-6.75
Subject 13	LH	10.2 ***	2550	-35.2	-12	-6.75
Mean LH				M = -34.74 SD = 4.88	M = -8.58 SD = 5.89	M = -8.74 SD = 9.19
Mean RH				M = 34.65 SD = 0.57	M = -7 SD = 2.16	M = -9.38 SD = 1.63

a Three sessions combined with four runs per session.

b Single session with four runs.

df indicates degrees of freedom in the first-level analysis (summedover sessions).

** p ≤ 0.01 uncorrected, ***p ≤ 0.001uncorrected.

### 3.2 Activity suppression in response to auditory stimuli

We found no voxel clusters responsive to the auditory condition after themultiple comparison correction in either hemisphere. Interestingly, we observeda significant cluster of voxels that showed a suppression of activity relativeto baseline during the auditory stimulation, but only in the right hemisphere(most significant voxel in cluster: z-statistic = -3.4, p < 0.001,d = 3.1, MNI x = 34.50, y = 0.75, z = -5.25, clustersize = 55.7 mm^3^,Fig.2a). There were no voxels suppressed by the auditory stimulation in theleft hemisphere even with a more liberal cluster-forming threshold of p <0.05. The auditory deactivations were not overlapping with visual activations(Fig. 2b).

### 3.3 Control analysis of auditory cortex activity

Since our main analysis did not reveal any significant activity in response toauditory stimulation that survived multiple comparisons correction, we ensuredthat our auditory stimuli were efficient in evoking auditory activity byanalyzing responses of the auditory cortex. We extracted the mean beta estimatesfor each condition (visual, auditory, audiovisual, and baseline) from thetransverse temporal gyrus (Heschl’s gyrus), which corresponds to theprimary auditory cortex. As expected, the primary auditory cortex exhibited nosignificant activation in response to visual stimuli (V-baseline, M =-0.84, SE = 0.25; t(14) = 0.24, p = 0.81, d = 0.02),but a significant activation in response to auditory stimuli (A-baseline, M= 2.13, SE = 0.62; t(14) = 5.4, p < 0.001, d= 1.92), to audiovisual stimuli (AV-baseline, M = 2.17, SE= 0.6; t(14) = 5.50, p < 0.001, d = 1.98) and whencomparing auditory and visual stimuli (V-A, t(14) = 5.23, p <0.001). These results are summarized inFigure2c. It is, therefore, unlikely that the absence of auditory activitywithin the claustrum is related to the inefficient auditory stimulation in ourexperiment.

### 3.4 No evidence for multisensory integration

We also looked at multisensory responses within the claustrum at the group level.We wanted to determine if there are claustrum regions beyond the unisensoryzones that show multisensory responses. Although we found a cluster that showedan activity pattern consistent with the superadditive response (AV +baseline) – (A + V), a closer examination of the cluster locationand the beta estimates for each condition revealed that first, the clusterlargely overlaps with the location of auditory deactivations and second, themultisensory contrast effects are driven by suppression of auditory responsesbelow the baseline (Supplementary Fig. 4). The latter is inconsistent with asuperadditive multisensory effect, which requires unisensory responses to behigher than the baseline (Beauchamp, 2005;Noppeney, 2012). We, therefore, didnot observe any signs of multisensory integration within the claustrum.

### 3.5 Session-to-session consistency of unisensory responses

Our main analysis thus reveals consistent bilateral visual responses at aspecific location within the human claustrum. To check whether the location ofvisual activity observed at the group level was consistent within individualsubjects across different scanning days, we performed a session-to-sessionconsistency analysis of visual activity, additionally including auditory effectsfor completeness. We used one session to define a group ofvisual/auditory-selective voxels and measured the responses of these same voxelsin the other session, statistically comparing the responses with zero.

**Fig. 3.**
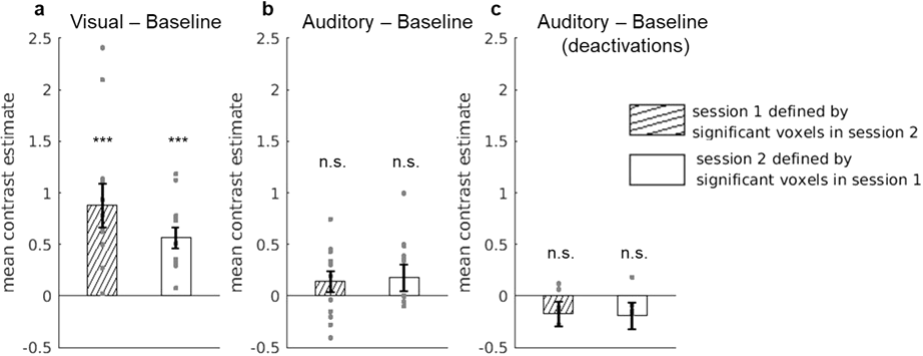
Consistency of visual and auditory responses between session 1 andsession 2. (a) Visual activity for each session shown as the meancontrast estimates for the V— baseline comparison (activation).(b) Auditory activity for each session shown as the mean contrastestimates for the A— baseline comparison (activation). (c)Auditory suppression for each session shown as the mean contrastestimates for the A— baseline comparison (deactivation for theright hemisphere only with N = 6). Error bars represent standarderror of the mean (Note that pilot subjects were not included in thisanalysis, yielding N = 12, for a & b.).***p < 0.001.

We found that visual responses were consistent from session-to-session, asindicated by above-zero contrast estimates for both sessions (Fig. 3a). A one-sample t-test comparing theeffects in each session against 0 revealed a significant effect for session 1(t(11) = 4.56, p < 0.001, d = 0.71) and a significanteffect for session 2 (t(11) = 4.86, p < 0.001, d = 0.59).In contrast, there was no significant auditory activity, neither for session 1(M = 0.21, SE = 0.12; t(11) = 1.69, p = 0.12, d= 0.22) nor for session 2 (M = 0.17, SE = 0.16; t(11)= 1.06, p = 0.31, d = 0.16) which is consistent with theabsence of auditory activations within the left and right claustrum at the grouplevel (Fig. 3b). For the auditorydeactivations, we looked at the sessions-to-session consistency only within theright hemisphere that showed significant deactivation effects at the group level(Fig. 3c). However, we could notconfirm the session-to-session consistency of these effects, as there was nosignificant difference from 0, neither for session 1 (M = -0.22, SE= 0.12; t(5) = -1.80, p = 0.13, d = 0.27), nor forsession 2 (M = -0.24, SE = 0.14; t(5) = -1.78, p =0.13, d = 0.29).

### 3.6 Behavioral performance

To ensure that participants were keeping their gaze on the fixation pointthroughout the experiment, we calculated percentage of hits and reaction times.As expected, we found a high average percentage of hits (M = 86.95%, SD= 17.93%, IQR = 79.96%— 97%) across all participants andreaction times were less than 1 second (M = 0.67 s, SD = 0.15 s,IQR = 0.65 s— 0.70 s).

## 4 Discussion

In this study, we aimed to investigate the visual, auditory, and audiovisual sensoryresponses within the human claustrum using ultra-high resolution 7T fMRI andnaturalistic video clips. We found visual responses within the claustrum that wereconsistent across sessions and appeared at a similar location across participants inboth hemispheres. We did not find significant auditory activity and no responsepattern consistent with audiovisual integration. These results provide the firstinsight into the modality-specific sensory responses within the human claustrum thathave otherwise been demonstrated only in animal models.

Our findings of visually evoked activity within the claustrum are in line with whatis known about claustrum physiology from animal models. For example, similar toRemedios et al. (2010)that foundvisually responsive neurons at the more ventral claustrum locations, we alsoobserved visual activity within a ventral claustrum site. Notably, a more ventrallocation of visual activity is also expected based on the topography ofclaustrocortical connections known from human tractography studies (Fernández-Miranda et al., 2008;Pathak & Fernandez-Miranda, 2014b). While thatstudy used natural movie clips, earlier studies using more controlled visualstimulation could also narrow down the feature-specific nature of the cells withinthe visual region of the cat claustrum (Sherk& LeVay, 1981). Neurons in the visual zone of the cat claustrumshowed preference for elongated moving bar stimuli as opposed to stationary stimuliand were particularly selective for the orientation of the bar stimuli. These animalmodel studies suggest that neurons in the visual claustrum zone may show apreference for specific features of the visual stimuli. We intentionally usednatural stimuli that contain a wide range of visual features. It, therefore, remainsunknown if particular features of the stimuli used may have evoked a strongerresponse in the visual zone of the claustrum compared to other features. Futureresearch that uses controlled manipulation of individual stimulus features (color,contrast, form, and motion) will have to determine what stimuli features the visualzone is more selective to in humans.

In contrast to our expectations based on primate literature (Remedios et al., 2014), we did not find any auditoryactivation within the claustrum in response to auditory stimulation. There may bedifferent reasons as to why auditory responses could not be found. Firstly, it islikely that the auditory zone is located in a thinner part of the claustrum comparedto the visual zone. The auditory zone identified, described in primates, was locatedmore dorsally compared to the visual region (Remedios et al., 2010). Naturally, the claustrum’s structurebecomes much thinner in the dorsal areas (Kapakin,2011). The auditory zone is thus expected to be more susceptible topartial volume effects and to produce a weaker functional signal, which we may havebeen unable to detect in the current experiment even using high spatial resolutionat 7 Tesla. Future investigations that aim to differentiate between the auditory andthe visual zone activity may require strategies that further increase the resolutionof functional images (Goense et al., 2016;Pohmann et al., 2016;Uğurbil, 2021;Viessmann & Polimeni, 2021).

Another potential reason for the lack of auditory response is the noise generated byimaging gradients, which could have attenuated the auditory activity. Althoughmeasures were taken to ensure that the auditory volume was loud enough forparticipants to hear the sounds despite the scanner noise, the overall level ofgradient noise may have led to a saturation of auditory activity, preventing us fromdetecting more subtle differences between the auditory stimulation and its absence.In an additional control analysis, we confirmed that our auditory stimuli evokedactivity in the auditory cortex. However, the corresponding effects in the claustrumcould have been smaller and thus harder to detect. Future studies aiming atmeasuring reliable auditory activity within the human claustrum could therefore takeadvantage of dedicated quiet EPI acquisition techniques that are tailored for fMRIstudies of auditory processing (De Martino et al.,2015;Peelle et al., 2010).

Finally, it is possible that the temporal pattern of auditory responses in theclaustrum neurons is different from that of the auditory cortex. A single-cellphysiology study investigating claustrum responses to natural vocalizations observedthat claustrum responses were highest when the vocalizations occurred immediatelyfollowing silence, which may point to the claustrum’s role in changedetection, an aspect closely related to attention (Remedios et al., 2014). Accordingly, the claustrum may only show aresponse in the auditory modality when there is a salient change from one stimulusto the next. In our experiment, the auditory stimuli, which consisted of naturalsounds (e.g., waterfalls, cat vocalizations, cowbells), may have not beensufficiently salient and behaviorally relevant to induce a response within theauditory claustrum. Future studies that focus on auditory processing within theclaustrum could test the role of saliency in evoking stronger auditory responsesusing, for example an auditory oddball paradigm.

Surprisingly, we found a deactivation in response to auditory stimuli in the rightclaustrum. This effect was observed in the right hemisphere only and was lessconsistent between sessions compared to visual activations. It should, therefore, beinterpreted with caution and followed up in future studies. At this point, we canonly speculate about the potential functional significance of these deactivations.One possibility is that deactivation in response to auditory stimuli mirrors thewell-known multisensory effects in the primary sensory cortices. It has beenrepeatedly shown that the presentation of stimuli in one modality leads to adeactivation in primary sensory cortex of the other modality (Driver & Noesselt, 2008;Gau et al., 2020). In this scenario, we would expectvisual activations and auditory deactivations to coincide spatially, which is notthe case (Fig. 2b). In addition, multisensoryeffects in the sensory cortices alone do not explain why deactivation only occurredin the right claustrum but not in the left one.

Another potential explanation is that these findings are due to attention-relatedprocessing within the claustrum and reflects distractor suppression, similar to whathas been described in rodent literature (Atlan etal., 2018;Goll et al., 2015). Inour case, the central fixation task was used primarily as a way to ensureparticipants were fixating their gaze at the center of the screen. However, it ispossible that because participants had to pay attention to the task, and hence tothe visual modality, auditory stimulation served as a distractor stimulus, leadingto suppression of the corresponding auditory representation within the claustrumwhenever auditory stimulation occurred. This idea is consistent with fMRI findingsin humans, which linked claustrum to within-modal and cross-modal divided attention(Vohn et al., 2007) and task control(Barrett et al., 2020) using conventionalfMRI.

Our study did not yield any evidence for multisensory responses within the claustrum.A previous study in primates also did not find any evidence of multisensoryresponses, at least in the visual and auditory claustrum zones (Remedios et al., 2010). Single-cell recordings as used inthat study are a unique possibility to measure activity of individual neurons withan unprecedented spatial and temporal resolution, but at the same time they limitthe spatial extent of brain tissue that can be examined within the same individual.Given the topography of the claustrocortical connections, multisensory responsescould reside outside of the sensory zones, for example in areas that project to orreceive inputs from the classical multisensory cortical regions such as thetemporo-parietal junction (TPJ) and the intraparietal sulcus (IPS) (Anderson et al., 2010;Geers & Coello, 2023;Ionta etal., 2011;Regenbogen et al.,2017). High-resolution functional MRI allowed us to measure activitythroughout the whole claustrum, yet we did not find any evidence for multisensoryeffects, even beyond the visual zone. As with the lack of auditory activity, wecannot entirely rule out that some subregion of the claustrum shows multisensoryresponses, but we were unable to detect them due to the limitations of our method.In future high-resolution studies, a detailed map of the claustrum’sconnection topography using other imaging modalities and techniques (resting-statefunctional MRI, DWI-based tractography) could be established to constrain the searchof multisensory responses to a more specific location within the claustrum.

Our study presents significant group-level claustrum activity, which is also evidentat the level of individual subjects and is consistent across two sessions for thesame subjects. Although this ensures the reliability and generalizability of ourresults in terms of the presence of visual effects (Editorial, 2020;P. L. Smith &Little, 2018), due to our moderately sized sample we are less confidentin our estimates of the underlying effect size (i.e., the magnitude of visualclaustrum activity). Therefore, future studies that intend to detect claustrumactivity and are interested in estimating its magnitude should consider conducing acareful a priori power analysis assuming medium effect sizes, rather than basingtheir sample planning on what is reported here (Poldrack et al., 2017).

Here, we present the first proof-of-concept study that has investigated thefine-scale functional response of the human claustrum using fMRI with high spatialresolution at ultra-high magnetic field. We demonstrate that it is possible todetect evoked visual activity within the human claustrum. Although further studiesare needed to determine the functional role of auditory deactivations, our currentresults for the visual modality open the possibility of studying theclaustrum’s contribution to visual processing.

## Supplementary Material

Supplementary Material

## Data Availability

Data and available scripts are provided at Code, and data are available on the OpenScience Framework (https://osf.io/7ebm2/).
