# Supplementary material for: High-resolution 7T fMRI reveals the visual zone of the human claustrum
Source: Imaging Neurosci (Camb). 2024 Oct 24;2:imag-2-00327. doi: 10.1162/imag_a_00327 (PMC12290541; doi:10.1162/imag_a_00327)
Supplement: Supplementary Material [file imag_a_00327-supp.pdf]

Title page

High-resolution 7T fMRI reveals the visual zone of the human claustrum – Supplementary Material

Adam Coates[1,2]*, David Linhardt[3], Christian Windischberger[3], Anja Ischebeck[1,2], Natalia Zaretskaya[1,2]

[1] Institute of Psychology, University of Graz, Graz, Austria
[2] BioTechMed, Graz, Austria
[3] High-Field MR Center, Center for Medical Physics and Biomedical Engineering, Medical University of Vienna, Vienna, Austria

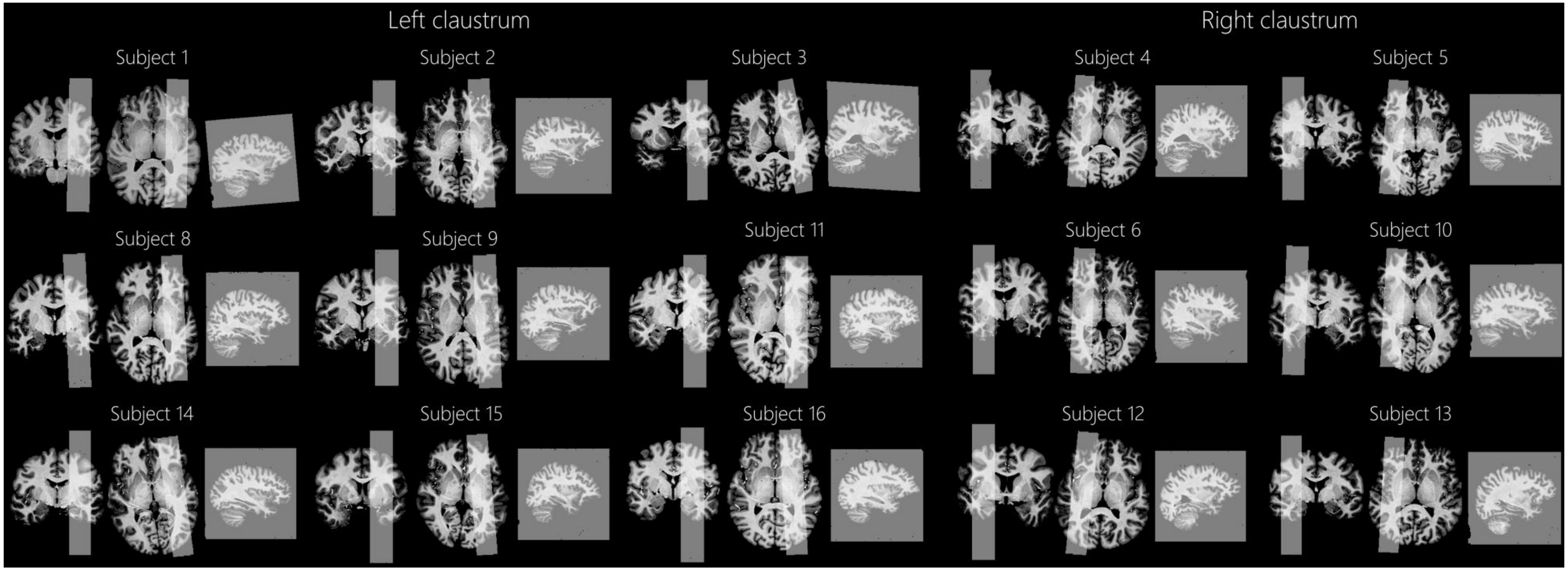

Supplementary Figure 1. Example field of view for the left and right claustrum for each subject.

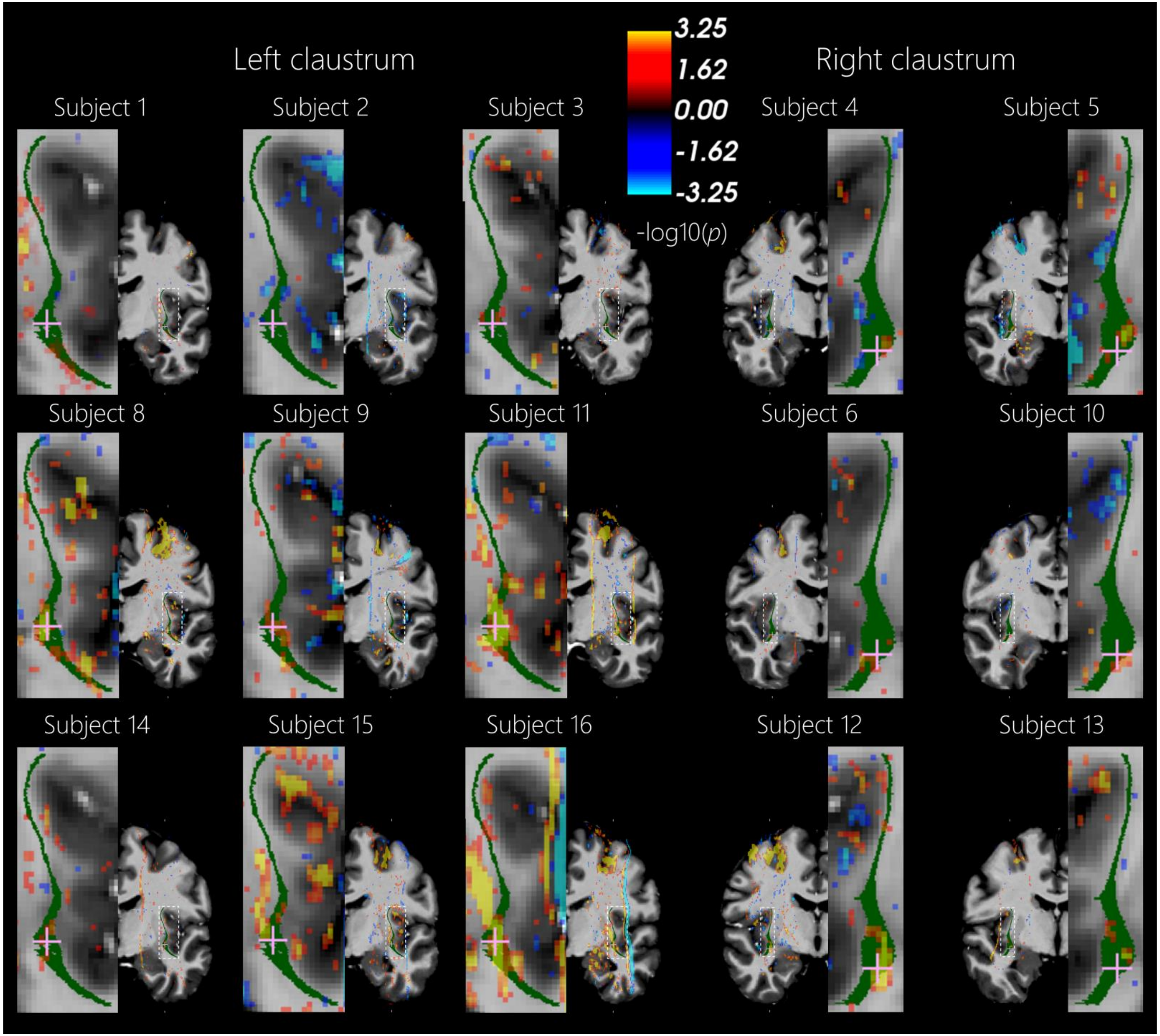

Supplementary Figure 2. The individual subject results, unmasked by the claustrum region of interest, are overlaid with each subject's anatomy in MNI space. Subject maps were thresholded on an individual basis depending upon the activity within the claustrum and are at least P < 0.05 (uncorrected). The center of mass of the significant cluster in the group results is shown as a crosshair. For the left claustrum, which showed two activation clusters in the group analysis, only the first (most significant) cluster is shown. Slices are zoomed-in on the claustrum ROI and do not represent the acquired field of view (see supplementary Figure 1 for the fields of view of every subject).

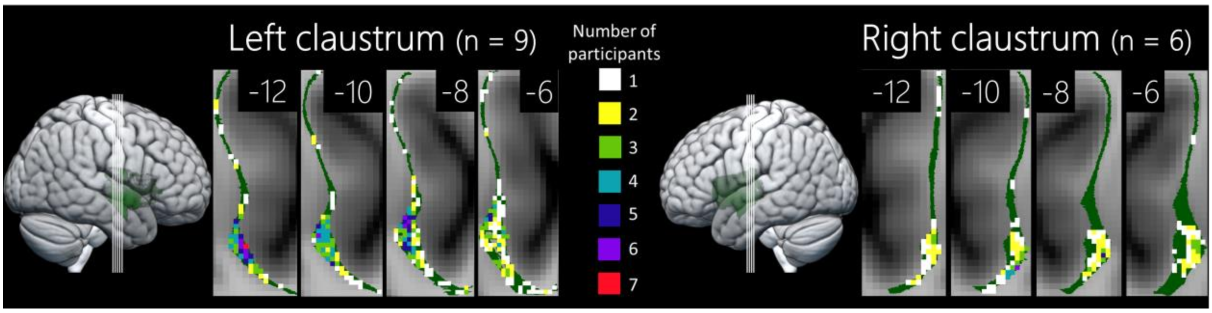

Supplementary Figure 3. Results of individual subjects overlaid in MNI space. Subject maps were binarized according to the significance level P < 0.05 (uncorrected) and are shown on an MNI template in coronal view. Color codes the number of participants with significant claustrum activity at each voxel. Slices are zoomed-in on the claustrum ROI and do not represent the acquired field of view (see supplementary Figure 1 for the fields of view of every subject).

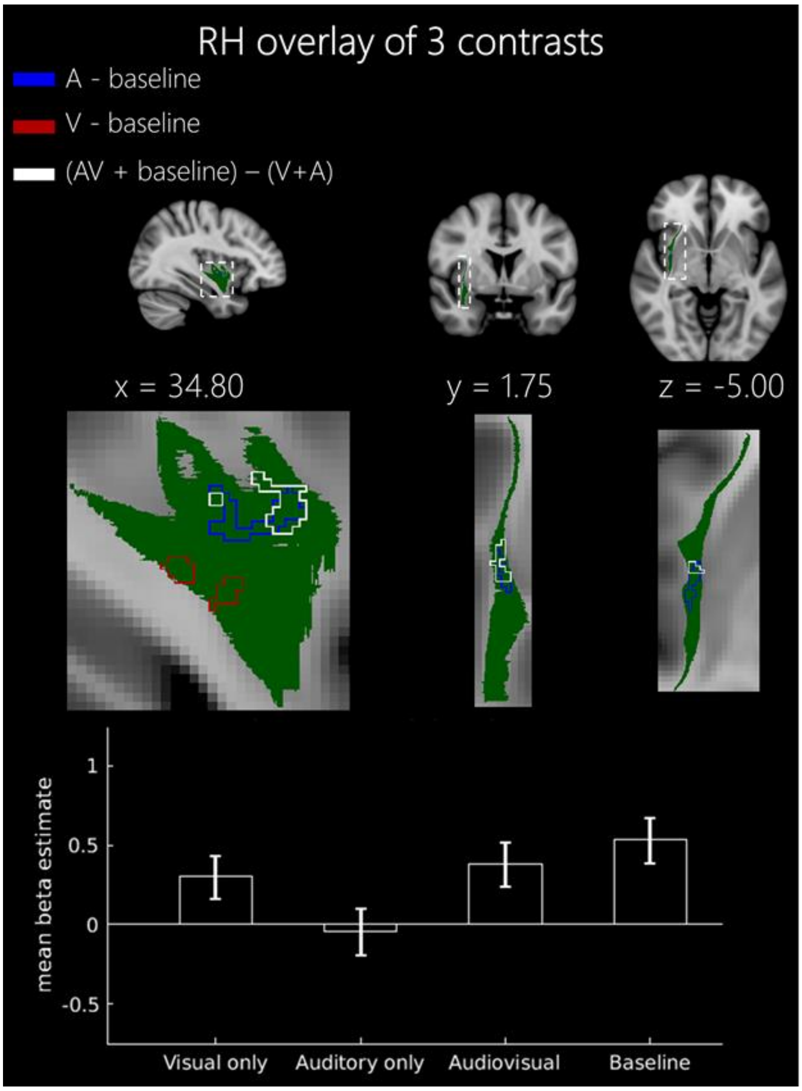

Supplementary Figure 4. Right claustrum auditory deactivations (blue), visual activations (red) and multisensory contrasts (white) overlayed onto the MNI template are shown as outlines to demonstrate the location and overlap of these effects. Bar plot represents the mean beta estimates for each experimental condition within the significant voxels of the right claustrum for the superadditive contrast (AV + baseline) – (V + A). Error bars represent SEM. Slices are zoomed-in on the claustrum ROI and do not represent the acquired field of view (see supplementary Figure 1 for the fields of view of every subject).
